# Periodicity and dosage optimization of an RNAi model in eukaryotes cells

**DOI:** 10.1186/s12859-019-2925-z

**Published:** 2019-06-17

**Authors:** Tongle Ma, Yongzhen Pei, Changguo Li, Meixia Zhu

**Affiliations:** 1 School of Computer Science and Technology Tianjin Polytechnic University, Tianjin, 300387 China; 2 School of Mathematical Sciences, Tianjin Polytechnic University, Tianjin, 300387 China; 3 Department of Basic Science, Army Military Transportation University, Tianjin, 300361 China

**Keywords:** RNA interference, Delay, Oscillation period, Sensitivity analyse, Optimal control

## Abstract

**Background:**

As a highly efficient and specific gene regulation technology, RNAi has broad application fields and good prospects. The effect of RNAi enhances as the dosage of siRNA increases, while an exorbitant siRNA dosage will inhibit the RNAi effect. So it is crucial to formulate a dose-effect model to describe the degradation effects of the target mRNA at different siRNA dosages.

**Results:**

In this work, a simple RNA interference model with hill kinetic function (Giulia Cuccato et al. (2011)) is extended. Firstly, by introducing both the degradation time delay *τ*_1_ of mRNA caused by siRNA and the transportation time delay *τ*_2_ of mRNA from the nucleus to the cytoplasm during protein translation, one acquires a novel delay differential equations (DDEs) model with physiology lags. Secondly, qualitative analyses are executed to identify regions of stability of the positive equilibrium and to determine the corresponding parameter scales. Next, the approximate period of the limit cycle at Hopf bifurcation points is computed. Furthermore we analyze the parameter sensitivity of the limit cycle. Finally, we propose an optimal strategy to select siRNA dosage which arouses significant silencing efficiency.

**Conclusions:**

Our researches indicate that when the dosage of siRNA is large, oscillating periods are identical for disparate number of siRNA target sites even if it greatly impacts the critical siRNA dosage which is the switch of oscillating behavior. Furthermore, parametric sensitivity analyses of limit cycle disclose that both of degradation lag and maximum degradation rate of mRNA due to RNAi are principal elements on determining periodic oscillation. Our explorations will provide evidence for gene regulation and RNAi.

## Background

The mechanism for sequence-specific post-transcriptional gene silencing that is induced by double-stranded RNA (dsRNA), leading to the regression of the target messenger RNA (mRNA) [1]. This common phenomenon in many eukaryotes, including insects, is named RNA interference (RNAi) by Fire et al. RNAi in animals [2] and in plants [3], is an evolutionarily conservative defense against transgenic or exotic virus infringement mechanism [4]. The process of RNAi can be divided into four stages:

- **Step 1.** Double stranded RNA (dsRNA) expressed in or introduced into the cell is cleaved into fragments of 21-23 base pairs (called small interfering RNA, abbreviated as siRNA) by the Dicer enzyme.
- **Step 2.** siRNAs are firstly adhered to RNA Induced Silencing Complex (RISC), and whereafter split into the sense strands which are deserted [5], and the antisense strands which are still roped to RISC.
- **Step 3.** An available siRNA-RISC complex, includes the siRNA loaded to the Ago protein, is packaged by antisense strand. Then it identifies and unites target mRNAs via the principle of complementary base pairing.
- **Step 4.** The antisense strand commands a endonuclease bound to RISC (an Argonaute protein called ‘slicer’) to operate the degradation of the target mRNA. And next the complex is liberated to dispose further mRNA targets.

In recent years, some studies have shown that synthetic siRNAs can effectively trigger RNAi in eukaryotes [6] and the siRNAs seem to avoid off-target effects prompted by longer double-stranded RNAs in mammalian cells [7]. This discovery makes the application of RNAi technology more convenient. The high efficiency and specificity of RNAi make it become a powerful tool for researching gene function. RNAi also provides a novel idea to schedule synthetic biological circuits for synthetic biology [8]. In the treatment of certain genetic diseases, for example viral infections [9], cancer [10] and inherited genetic disorders [11], RNAi has the potential to become a new type of therapeutic tool. In the field of pest management, RNAi also shows its talents [12]. And RNAi technology first approved by the US Environmental Protection Agency as a pesticide in 2017.

Because excessive siRNAs not only affect its efficiency [13], but attract off-target effect [7]. For RNAi application, it is necessary to find a quantitative mathematical model that can describe the relationship between the dosage of siRNA and the RNAi effect. Giulia Cuccato et al. (2011), according to vitro experimental data and squared error measure, capture the most efficient mathematical model of RNA interference in [13].

However, for the model, we consider that there are two important time delays that cannot be ignored during the entire RNAi process. First, degradation of mRNA due to RNAi. Here, we use *τ*_1_ to describe this time delay. Next, carriage of mRNA from nucleus to cytoplasm. Thus, we introduce *τ*_2_ to represent this time delay. In our work, we start from the model proposed in [13] and then modify it. First, we conduct a qualitative analysis of the model with delay. Our result show that the stability of the only positive equilibrium has changed: it is stable while the original model without time delays, as the time delay increases, it will turn into damped oscillation and lose its stability via a Hopf Bifurcation. Therefore, time delay plays an important role in dynamics of RNAi model and should not be ignored in the modeling of genetic regulation. Next, we introduce the solution to the periodic value of the periodic solution of the system with the limit cycle. And we analyze the parameter sensitivity of the amplitude and period of a periodic solution for a system with a limit cycle. Finally, we give optimal control for quantitative RNAi model by optimization theory.

## Results

### Qualitative analysis

When the delays are finite, the characteristic equations are functions of delays. As values of the delays change, the stability of the trivial solution may also changes. Such phenomena is often refereed to as stability switches. Next the qualitative analysis of model (20) will be conducted.

#### Stability and Hopf Bifurcation

In this section, we discuss the local asymptotic stability of the unique positive equilibrium $Q^{*}(\tilde M, \tilde P)$ and the existence of Hopf bifurcation. Setting *β*=*rS*^*n*^/(*θ*^*n*^+*S*^*n*^), $\tilde M $ and $\tilde P$ are denoted by

$$\tilde M=\frac{k_{m}}{d_{m}+\beta},~~~~~~\tilde P=\frac{k_{p}}{d_{p}}\tilde{M}. $$

For *τ*_1,2_>0, characteristic equation of model (20) is given by

1 $$ \begin{array}{l} (d_{m}+\beta e^{-\lambda\tau_{1}}+\lambda)(d_{p}+\lambda)=0. \end{array}  $$

Obviously, *λ*_1_=−*d*_*p*_ is a negative root of the Eq. (1). Next let the first item of the left side of the Eq. (1) be

2 $$ \begin{array}{l} f(\lambda)=d_{m}+\beta e^{-\lambda\tau_{1}}+\lambda. \end{array}  $$

##### **Lemma 1**

For *ω*∈[*π*/(2*τ*_1_),*π*/*τ*_1_], let $\phantom {\dot {i}\!}\beta _{0}=e^{-d_{m}\tau _{1}-1}/\tau _{1},$ *β*_1_=−*d*_*m*_/ cos(*ωτ*_1_)>0. Then the following results hold.

(*a*) If *β*<*β*_0_, *f*(*λ*) has two real negative roots.

(*b*) If *β*=*β*_0_, *f*(*λ*) has one real negative root.

(*c*) If *β*_0_<*β*<*β*_1_, *f*(*λ*) has two complex conjugate roots with *Re*(*λ*)<0.

(*d*) If *β*=*β*_1_, *f*(*λ*) has two complex conjugate roots with *Re*(*λ*)=0.

(*e*) If *β*>*β*_1_, *f*(*λ*) has two complex conjugate roots with *Re*(*λ*)>0.

##### *Proof*

Function (2) implies *f*(−*∞*)=+*∞*, *f*(+*∞*)=+*∞* and *f*(0)=*d*_*m*_+*β*>0. Then, letting $\phantom {\dot {i}\!}f^{\prime }(\lambda)=1-\beta \tau _{1}e^{-\lambda \tau _{1}}=0$ yields

$$\lambda^{*}=\frac{1}{\tau_{1}}\ln(\beta\tau_{1}). $$

So, *f*(*λ*) maybe has negative root only if *βτ*_1_<1. In addition, because *f*(*λ*^∗^) is minimum of *f*(*λ*) for every $\lambda \in \mathbb {R}$, thus the function (2) has one real negative root *λ*^∗^ if *f*(*λ*^∗^)=0, namely, *β*=*β*_0_. Hence (*b*) is proved. If *β*<*β*_0_, we obtain *f*(*λ*^∗^)<0 and the function (2) has two negative real roots, then (*a*) is proved too.

For *β*>*β*_0_, the Eq. (2) may has two complex roots. For that, we assume that there exists a solution of the characteristic equation of the form *λ*=*iω*(*ω*>0). Putting it into *f*(*λ*), it follows

$$\begin{array}{l} d_{m}+\beta\cos(\omega\tau_{1})-\beta i\sin(\omega\tau_{1})+i\omega=0. \end{array} $$

Comparing real and imaginary parts we get,

3 $$ \begin{array}{ll} \cos(\omega\tau_{1})=-\frac{d_{m}}{\beta},~~~ \sin(\omega\tau_{1})=\frac{\omega}{\beta}.\vspace{0.5em} \end{array}  $$

Squaring and adding the first and the second of (3), we get $(d_{m}^{2}+\omega ^{2})/\beta ^{2}=1$, that is, $\omega ^{2}=\beta ^{2}-d_{m}^{2}$. Hence positive solution $\omega _{0}=\sqrt {\beta ^{2}-d_{m}^{2}}$ exists if *β*>*d*_*m*_. And, corresponding to *λ*=*iω*_0_ and the first equation of (3), there exists $\tau _{1}^{*}>0$ such that,

$$\begin{array}{l} \tau_{1}^{*}=\frac{1}{\omega_{0}}\arccos\left(-\frac{d_{m}}{\beta}\right),\\ \beta_{1}=-\frac{d_{m}}{\cos(\omega_{0}\tau_{1})}, \qquad \omega_{0}\in[\pi/(2\tau_{1}),\pi/\tau_{1}], \end{array} $$

and (*c*) and (*d*) are proved. When *β*>*β*_1_, *Re*(*λ*)>0, so (*e*) is proved. □

##### **Theorem 1**

For model (20), the following results hold.

(*a*) If *β*≤*β*_0_, then the equilibrium *Q*^∗^ is asymptotically stable.

(*b*) If *β*_0_<*β*<*β*_1_, then the equilibrium *Q*^∗^ is oscillatory stable.

(*c*) If *β*>*β*_1_, then the equilibrium *Q*^∗^ is unstable. Furthermore if *β*=*β*_1_, Hopf bifurcation occurs.

##### *Proof*

(*a*) and (*b*) are apparently valid by Lemma 1. Now differentiating (1) with respect to *τ*_1_ gives

$$ \begin{array}{lll} \left(\frac{d\lambda}{d\tau_{1}}\right)^{-1}&=\frac{2\lambda+\beta d_{p}e^{-\lambda\tau_{1}}(-\tau_{1})+\beta e^{-\lambda\tau_{1}}+\beta\lambda e^{-\lambda\tau_{1}}(-\tau_{1})+d_{m}+d_{p}}{\beta\lambda e^{-\lambda\tau_{1}}(d_{p}+\lambda)} \vspace{0.5em} \\ &=\frac{2\lambda+d_{m}+d_{p}}{\beta\lambda e^{-\lambda\tau_{1}}(d_{p}+\lambda)}-\frac{\tau_{1}}{\lambda}+\frac{1}{\lambda(d_{p}+\lambda)} \vspace{0.5em} \\ &= \frac{2\lambda+d_{m}+d_{p}}{\lambda(-\lambda^{2}-(d_{m}+d_{p})\lambda-d_{m}d_{p})}-\frac{\tau_{1}}{\lambda}+\frac{1}{\lambda(d_{p}+\lambda)}. \end{array} $$

Then at *λ*=*iω*_0_, one gets

$$ \begin{array}{llll} \left(\frac{d\lambda}{d\tau_{1}}\right)^{-1}\bigg|_{\lambda=i\omega_{0}}&=\frac{2i\omega_{0}+d_{m}+d_{p}}{i\omega_{0}(\omega_{0}^{2}-(d_{m}+d_{p})i\omega_{0}-d_{m}d_{p})}\,-\,\frac{\tau_{1}}{i\omega_{0}}\,+\,\frac{1}{d_{p}i\omega_{0}-\omega_{0}^{2}} \\ &=\frac{(d_{m}+d_{p})^{2}\omega_{0}^{2}+2\omega_{0}(\omega_{0}^{3}-d_{m}d_{p}\omega_{0})}{((d_{m}+d_{p})\omega_{0}^{2})^{2}+(\omega_{0}^{3}-d_{m}d_{p}\omega_{0})^{2}}-\frac{1}{d_{p}^{2}+\omega_{0}^{2}} \\ &\quad+\frac{2(d_{m}+d_{p})\omega_{0}^{3}+(d_{m}+d_{p})(\omega_{0}^{3}-d_{m}d_{p}\omega_{0})}{((d_{m}+d_{p})\omega_{0}^{2})^{2}+(\omega_{0}^{3}-d_{m}d_{p}\omega_{0})^{2}}i \\ &\quad+\frac{\tau_{1}}{\omega_{0}}i-\frac{d_{p}}{\omega_{0}(d_{p}^{2}+\omega_{0}^{2})}i. \end{array} $$

Thus

$${\begin{aligned} \begin{array}{lll} \left(\frac{dRe\lambda(\tau_{1}^{*})}{d\tau_{1}}\right)^{-1}\bigg|_{\lambda=i\omega_{0}}&=\frac{(d_{m}+d_{p})^{2}\omega_{0}^{2}+2\omega_{0}(\omega_{0}^{3}-d_{m}d_{p}\omega_{0})}{((d_{m}+d_{p})\omega_{0}^{2})^{2}+(\omega_{0}^{3}-d_{m}d_{p}\omega_{0})^{2}}-\frac{1}{d_{p}^{2}+\omega_{0}^{2}} \\ &=\frac{\omega_{0}^{2}((d_{m}+d_{p})^{2}d_{p}^{2}+(\omega_{0}^{2}-d_{m}d_{p})(2d_{p}^{2}+\omega_{0}^{2}+d_{m}d_{p}))}{(((d_{m}+d_{p})\omega_{0}^{2})^{2}+(\omega_{0}^{3}-d_{m}d_{p}\omega_{0})^{2})(d_{p}^{2}+\omega_{0}^{2})} \vspace{0.5em} \\ &=\frac{\omega_{0}^{2}(\omega_{0}^{4}+2\omega_{0}^{2}d_{p}^{2}+d_{p}^{4})}{(((d_{m}+d_{p})\omega_{0}^{2})^{2}+(\omega_{0}^{3}-d_{m}d_{p}\omega_{0})^{2})(d_{p}^{2}+\omega_{0}^{2})}>0\vspace{0.5em}, \end{array} \end{aligned}} $$

$$\begin{array}{l} sign\left(\frac{dRe\lambda(\tau_{1}^{*})}{d\tau_{1}}\right)=sign\left[\left(\frac{dRe\lambda(\tau_{1}^{*})}{d\tau_{1}}\right)^{-1}\right]=1. \end{array} $$

□

So, *Re*(*λ*)>0 providing $\tau _{1}>\tau _{1}^{*}$. By the Hopf bifurcation theorem, the condition with $\tau _{1}>\tau _{1}^{*}$ and *Re*(*λ*)>0 guarantees that the Hopf bifurcation at *β*=*β*_1_ is supercritical. The result (*c*) is proved.

**Fig. 1:**
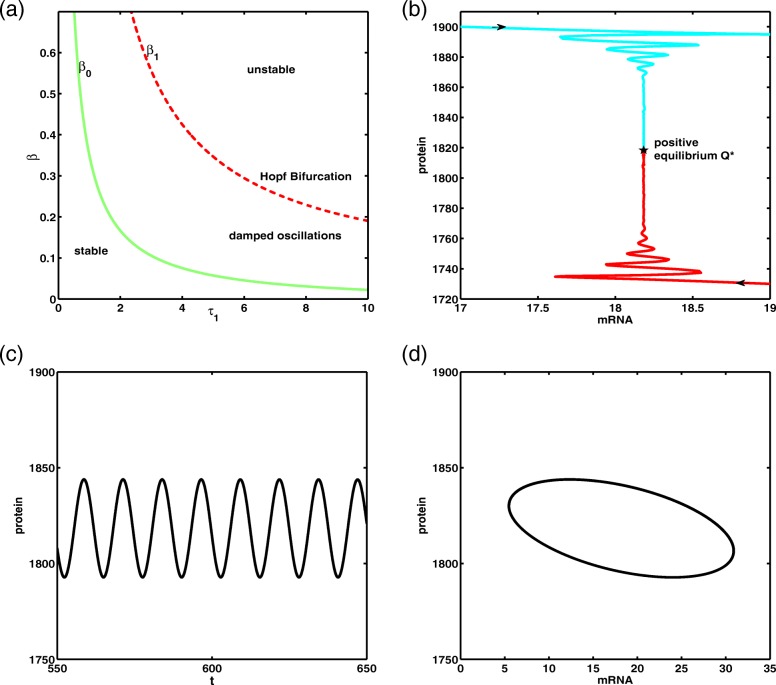
(*a*) Bifurcation diagram for delay model. The functions *β*_0_(*τ*_1_) and *β*_1_(*τ*_1_) are defined in “Qualitative analysis” section. In the region marked ‘stable’, the positive equilibrium *Q*^∗^ is stable. In the region marked ‘damped oscillations’, the solutions of the delay model converge steadily to the positive equilibrium as *t* is large enough. At Hopf Bifurcation value, the solutions of the delay model converge consistently to limit cycle. (*b*) Phase diagram of delay model with parameters *r*=0.5062, *τ*_1_=2.5 and *τ*_2_=1 corresponding to ‘damped oscillations’ region. (*c*) Sustained oscillations of protein at Hopf Bifurcation value *τ*_1_=3.3594. (*d*) Phase diagram of delay model at Hopf Bifurcation value *τ*_1_=3.3594

The bifurcation diagram of Eq. (20) as a function of the delay *τ*_1_ and of the parameter *β* is shown in Fig. 1a. Using some parameter values suggested in [14], we take *k*_*m*_=10, *d*_*m*_=0.05, *k*_*p*_=1, *d*_*p*_=0.01, other parameter values are set by *θ*=10, *n*=4, *S*=30. It is always possible to choose values of *r*, *θ*, *n* and *S* such that *β*>*β*_1_(*τ*_1_), where *β*_1_(*τ*_1_) is the parameter that determines a supercritical Hopf Bifurcation. In this case, the delay model (20) has asymptotically stable oscillatory solutions (limit cycle solutions) in Fig. 1a, and the time evolution of protein is shown in Fig. 1c. The phase diagrams for system (20) in damped oscillation region and at the limit cycle are shown in Fig. 1b and d, respectively.

#### Period of the bifurcating oscillatory solution

We knew in the above subsection how the delay differential equation model was capable of generating limit cycle periodic solutions. One indication of their existence is if the steady state is unstable by growing oscillations, although this is certainly not conclusive. From the analysis of the previous section, we found that *τ*_2_ has no effect on the stability of the system, and the first equation of (20) is independent. So, resorting to the periodicity of the first equation, we intent to analyze the period of the whole system as the delay occurs. Hence pull out the first equation of (20) separately,

4 $$ \begin{array}{l} \frac{dM(t)}{dt}=k_{m}-d_{m}M(t)-\frac{rS^{n}}{\theta^{n}+S^{n}}M(t-\tau_{1}). \end{array}  $$

Linearising (4) at the first component of the steady state, $\tilde {M}=k_{m}/(d_{m}+\beta)$, that is, writing $M(t)-\tilde {M}=m(t)$, yields

5 $$ \begin{array}{l} \frac{dm(t)}{dt}=-d_{m}m(t)-\beta m(t-\tau_{1}). \end{array}  $$

By looking for solutions *m*(*t*) in the form *m*(*t*)=*ce*^*λt*^, we get

6 $$ \begin{array}{l} \lambda=-d_{m}-\beta e^{-\lambda\tau_{1}}, \end{array}  $$

where *c* is a constant and the eigenvalues *λ* are solutions of (6), a transcendental equation in which *τ*_1_>0. It is not easy to find the analytical solutions of (6). However, all we really want to know from a stability point of view is whether there are any solutions with *Re*(*λ*)>0 which from the form of *m*(*t*) implies instability since in this case *m*(*t*) grows exponentially with time.

Putting *λ*=*μ*+*iω*, in (6), and now take the real and imaginary parts of the transcendental equation in (6), namely,

7 $$ \begin{array}{l} \mu=-d_{m}-\beta e^{-\mu\tau_{1}}\cos\omega\tau_{1},\qquad\omega=\beta e^{-\mu\tau_{1}}\sin\omega\tau_{1}. \end{array}  $$

We knew that the steady state *Q*^∗^ is stable if $0<\tau _{1}<\tau _{1}^{*}$ and the delay Eq. (20) has an stable periodic solution for $\tau _{1}=\tau _{1}^{*}$. In the latter case we expect the solution to exhibit stable limit cycle behaviour. The critical value $\tau _{1}=\tau _{1}^{*}$ is the bifurcation value. The effect of delay in models is usually to increase the potential for instability. Here as *τ*_1_ is increased beyond the bifurcation value $\tau _{1}^{*}$, the steady state becomes unstable.

Near the bifurcation value we can get a estimate of the period of the bifurcating oscillatory solution as follows. Consider the dimensionless form and let

8 $$ \begin{array}{l} \tau_{1}=\tau_{1}^{*}+\varepsilon,\qquad 0<\varepsilon\ll1. \end{array}  $$

The solution *λ*=*μ*+*iω*, of (7), with the *Re*(*λ*)=0 when $\tau _{1}=\tau _{1}^{*}$ is *μ*=0, $\omega =\omega _{0}=\sqrt {\beta ^{2}-d_{m}^{2}}$. For *ε* small we expect *μ* and *ω* to differ from *μ*=0 and *ω*=*ω*_0_ also by small quantities so let

9 $$ \begin{array}{l} \mu=\delta,\quad\omega=\omega_{0}+\sigma,\quad0<\delta\ll1,\qquad\left|\sigma\right|\ll1, \end{array}  $$

where *δ* and *σ* are to be determined. Substituting these into the second of (7) and expanding for small *δ*, *σ* and *ε* gives

$$\begin{array}{l} \omega_{0}+\sigma=\beta e^{-\delta(\tau_{1}^{*}+\varepsilon)}\sin[(\omega_{0}+\sigma)(\tau_{1}^{*}+\varepsilon)]\Rightarrow\ \\ \qquad\,\,\sigma\approx -d_{m}\sigma\tau_{1}^{*}-d_{m}\varepsilon\omega_{0}-\omega_{0}\delta\tau_{1}^{*}, \end{array} $$

while the first of (7) gives

$$ \begin{array}{l} \delta=-d_{m}-\beta e^{-\delta(\tau_{1}^{*}+\varepsilon)}\cos[(\omega_{0}+\sigma)(\tau_{1}^{*}+\varepsilon)]\Rightarrow\ \\ \delta\approx \omega_{0}\sigma\tau_{1}^{*} +\omega_{0}^{2}\varepsilon-d_{m}\delta\tau_{1}^{*}. \end{array} $$

Thus on solving these simultaneously

$$\begin{array}{l} \sigma\approx \frac{-(1+d_{m}\tau_{1}^{*})d_{m}\varepsilon\omega_{0}-\omega_{0}^{3}\tau_{1}^{*}\varepsilon}{(1+d_{m}\tau_{1}^{*})^{2}+\omega_{0}^{2}{\tau_{1}^{*}}^{2}}. \end{array} $$

Considering that the imaginary part of *λ* is the root cause of *m*(*t*) periodicity, near the bifurcation, the first of (5) with (6) gives

$${\begin{aligned} \begin{array}{ll} M(t)&=\tilde{M}+Re\left\{c\exp[\delta t+i(\omega_{0}+\sigma)t]\right\} \vspace{0.5em} \\ &\approx \tilde{M}\!+Re\left\{c\exp(\delta t)\exp[it(\omega_{0}\,-\,\frac{(1+d_{m}\tau_{1}^{*})d_{m} \varepsilon\omega_{0}+\omega_{0}^{3}\tau_{1}^{*}\varepsilon}{(1+d_{m}\tau_{1}^{*})^{2}+\omega_{0}^{2}{\tau_{1}^{*}}^{2}})]\!\right\}. \end{array} \end{aligned}} $$

This shows that the delay Eq. (20) has an stable periodic solution due to the occurrence of the Hopf bifurcation with period

$$ \begin{array}{l} T= \frac{2\pi}{\omega_{0}-\frac{(1+d_{m}\tau_{1}^{*})d_{m}\varepsilon\omega_{0}+\omega_{0}^{3}\tau_{1}^{*}\varepsilon}{(1+d_{m}\tau_{1}^{*})^{2}+ \omega_{0}^{2}{\tau_{1}^{*}}^{2}}} \approx \frac{2\pi}{\omega_{0}}=\frac{2\pi}{\arccos(-\frac{d_{m}(\theta^{n}+S^{n})}{rS^{n}})}\tau_{1}^{*}. \end{array} $$

**Fig. 2:**
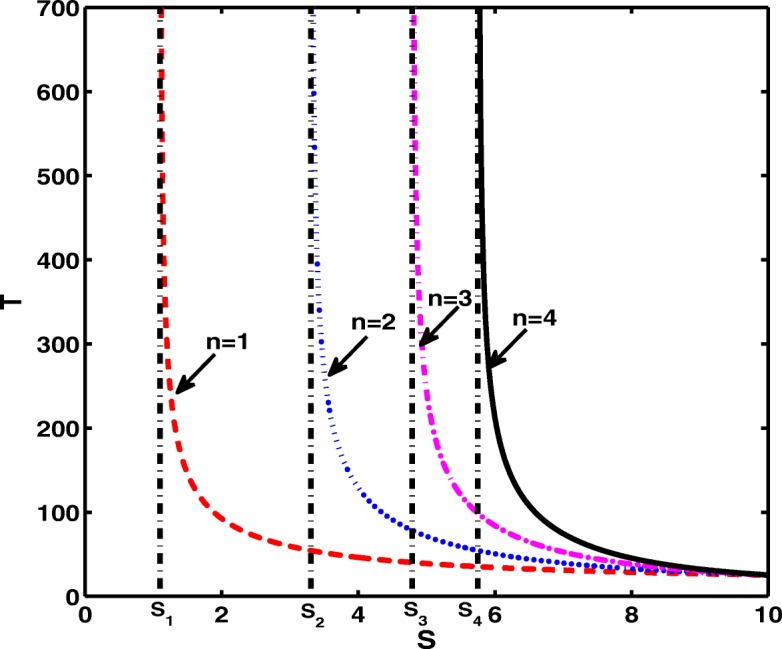
Relationship among period *T*, siRNA dosage *S* and the number of siRNA target sites *n*

*T* describes the relations of the Hopf bifurcation period with the maximal regression rate *r*, siRNA dosage *S*, the half saturation coefficient *θ* and the number of siRNA target sites *n*. Here, Fig. 2 reveals that the levels of mRNA and the protein are persistence when the dosage of siRNA are small, otherwise the periodic oscillating happens. Meanwhile, it indicates that when the dosage of siRNA is large, oscillating periods are identical for disparate number of siRNA target sites even though it greatly impacts the critical siRNA dosage *S*_*n*_ which is the switch of oscillating behavior.

Our delayed differential equations are applied to model gene regulatory network due to RNAi. The periodic solutions of delayed differential equations are subjected to parameters. So, it is necessary that a parametric sensitivity analysis for amplitude and period of periodic solutions.

### Parametric Sensitivity

In this section, we use a sensitivity analysis method proposed in [15], and focus on sensitivities of amplitude and of the period when our delay model (20) possesses a periodic solution. Then define the sensitivity equation for parameter *d*_*m*_:

$$\left\{ \begin{array}{ll} \frac{dR_{d_{m}}^{M}(t)}{dt}=-d_{m}R_{d_{m}}^{M}(t)-\frac{rS^{n}}{S^{n}+\theta^{n}}R_{d_{m}}^{M}(t-\tau_{1})-M(t), \\ \frac{{dR}_{d_{m}}^{P}(t)}{dt}=-d_{p}R_{d_{m}}^{P}(t)+k_{p}R_{d_{m}}^{M}(t-\tau_{2}) \vspace{0.5em}. \end{array}\right. $$

In similar way, we get the sensitivity equation with respect to *τ*_1_:

$${\begin{aligned} \left\{\begin{array}{lll} \frac{{dR}_{\tau_{1}}^{M}(t)}{dt}&= -d_{m}R_{\tau_{1}}^{M}(t)-\frac{rS^{n}}{S^{n}+\theta^{n}}[R_{\tau_{1}}^{M}(t-\tau_{1})-(k_{m}-d_{m}M(t-\tau_{1}) \vspace{1em}\\ &\quad-\frac{rS^{n}}{S^{n}+\theta^{n}}M(t-2\tau_{1}))], \vspace{0.5em} \\ \frac{{dR}_{\tau_{1}}^{P}(t)}{dt}&= -d_{p}R_{\tau_{1}}^{P}(t)+k_{p}R_{\tau_{1}}^{M}(t-\tau_{2}).\vspace{0.5em} \end{array}\right. \end{aligned}} $$

**Fig. 3:**
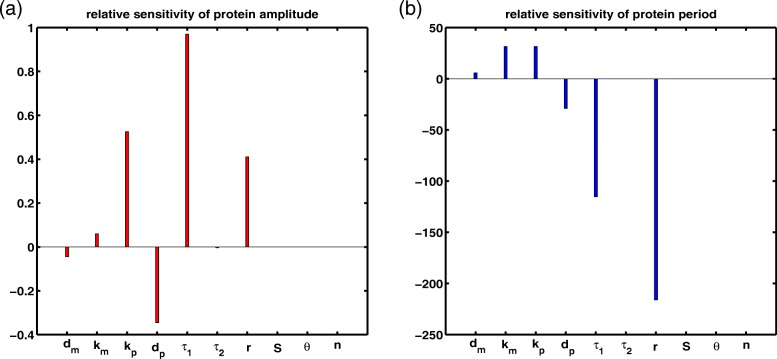
Relative sensitivities of the amplitude (a) and of period (b) in the dosage of protein. Nominal parameter values: *k*_*m*_=10, *d*_*m*_=0.05, *r*=0.5062, *S*=30, *θ*=10, *n*=4, *k*_*p*_=1, *d*_*p*_=0.01, *τ*_1_=3.3594, *τ*_2_=1

Analogously, the other sensitivity equations on the rest parameters can be captured, I won’t list them here. Solving the there equations, and according to the circumscription of sensitivities of the limit cycle in [15], we obtain the relative sensitivities of the amplitude and of the period shown in Fig. 3.

We observe that *τ*_1_, RNAi process delay, has a effective impact on both amplitude and period, while *τ*_2_, the mRNA translational delay, has inappreciable influence. Because, the occurrence of the limit cycle is only related to the value of *τ*_1_, and *τ*_2_ does not affect the stability of the equilibrium point of model (20). Moreover, parameter *r*, the maximal degradation rate of the mRNA due to RNAi, has a important affection on period too. This is because the value of *r* determines the satisfaction of *β*=*β*_1_. When *β*=*β*_1_, the system (20) will have a limit cycle, where *β* is the degradation rate of mRNA due to RNAi. In other words, in eukaryotic cells, if the rate of degradation of mRNA due to RNAi is greater than the rate of degradation of the mRNA itself, *τ*_1_ and *r* will be important parameters in the quantitative delay system (20).

### Optimizing the dosage of siRNA in RNAi

During the RNAi, excessive siRNAs not only affect the efficiency, but also attracts off-target effect. So the rational dosage of siRNA is crucial for both enhancing RNAi efficiency and reducing cost.

#### Optimal control for model without delay

Define a cost function as

10 $$ \begin{array}{l} J=P_{s}ST+\int_{0}^{T} P(t) \,dt, \end{array}  $$

where *P*_*s*_ is the cost of per unit siRNA, *T* is the terminal time. The first part of (10) represents the cost of siRNA consumed in [0,*T*], and the second part shows the accumulation of protein (denoted by PA) in [0,*T*]. The aim of this work is to minimize the cost of siRNA and the accumulation of protein.

**Problem(Q1).** For model (19), choose *S*∈[0,200] (according to the experiments in [13]) to minimize the cost function (10).

Since the constraint of the cost function (10) is only the state equation, it must be observed during [0,*T*]. Then the Lagrange multiplier vector can be used to introduce the equality constraint into the integrand part of the definite integral, thus transforming the constrained optimization problem into an unconstrained optimization problem. Then there is

11 $$ {\begin{aligned} \begin{array}{ll} \tilde{J}&= P_{s}ST+\int_{0}^{T}P(t)+\lambda_{1}\left(k_{m}-d_{m}M(t)-r\frac{S^{n}}{\theta^{n}+S^{n}}M(t)-\dot{M}(t)\right) \\ &\quad+\lambda_{2}\left(k_{p}M(t)-d_{p}P(t)-\dot{P}(t)\right)dt \vspace{0.5em}. \end{array} \end{aligned}}  $$

Introduce a Hamiltonian function *H* as follows:

$$\begin{array}{l} H=P(t)+\lambda_{1}\left(k_{m}-d_{m}M(t)-r\frac{S^{n}}{\theta^{n}+S^{n}}M(t)\right)\\ \quad\quad+\lambda_{2}\left(k_{p}M(t)-d_{p}P(t)\right). \end{array} $$

Then corresponding costate equations is determined by

12 $$ {\left\{ \begin{array}{lll} \dot{\lambda}_{1}(t)=-\frac{\partial H}{\partial M(t)}=\lambda_{1}(t)(d_{m}+r\frac{S^{n}}{\theta^{n}+S^{n}})-\lambda_{2}k_{p}, \vspace{0.5em} \\ \dot{\lambda}_{2}(t)=-\frac{\partial H}{\partial P(t)}=\lambda_{2}(t)d_{p}-1, \vspace{0.5em} \\ \lambda_{1}(T)=0,\quad \lambda_{2}(T)=0, \vspace{0.5em} \end{array}\right.}  $$

and the corresponding gradients of the cost function (11) with respect to *S* is

13 $$ \begin{array}{l} \frac{\partial\tilde{J}(S)}{\partial S}=P_{s}T-\int_{0}^{T} \lambda_{1}M(t)r\theta^{n}\frac{nS^{n-1}}{(\theta^{n}+S^{n})^{2}}dt. \end{array}  $$

#### Optimal control for model with delay

**Problem (Q2)** For model (20), opt for *S*∈[0,200] (according to the experiments in [13]) to minimize the cost function (10).

Rewriting the cost function (10) in the same way, yields

14 $$ \begin{aligned} \begin{array}{ll} \tilde{J}=& P_{s}ST+\int_{0}^{T}P(t)+\lambda_{1}\\&\left(k_{m}-d_{m}M(t)-r\frac{S^{n}}{\theta^{n}+S^{n}}M(t-\tau_{1})-\dot{M}(t)\right) \vspace{0.5em} \\ &+\lambda_{2}\left(k_{p}M(t-\tau_{2})-d_{p}P(t)-\dot{P}(t)\right)dt \vspace{0.5em}. \end{array}\end{aligned}  $$

Define a Hamiltonian function *H* by

$$\begin{array}{ll} H&=P(t)+\lambda_{1}\left(k_{m}-d_{m}M(t)-r\frac{S^{n}}{\theta^{n}+S^{n}}M(t-\tau_{1})\right)\vspace{0.5em}\\ &\quad+\lambda_{2}\left(k_{p}M(t-\tau_{2})-d_{p}P(t)\right). \end{array} $$

The corresponding costate equations is dominated by

15 $$ \left\{ \begin{array}{ll} \dot{\lambda_{1}}(t)= d_{m}\lambda_{1}(t)+r\frac{S^{n}}{\theta^{n}+S^{n}}\lambda_{1}(t+\tau_{1})-k_{p}\lambda_{2}(t+\tau_{2}), \vspace{0.5em} \\ \dot{\lambda_{2}}(t)=d_{p}\lambda_{2}(t)-1, \vspace{0.5em} \end{array}\right.  $$

with jump conditions

16 $$ \begin{array}{l} \lambda_{1}(T^{-})=\lambda_{1}(T^{+}),~~~\lambda_{2}(T^{-})=\lambda_{2}(T^{+}), \end{array}  $$

and boundary conditions

17 $$ \begin{array}{l} \lambda_{1}(t)=0,~~~\lambda_{2}(t)=0,~~~t\geq T. \end{array}  $$

The corresponding gradients of the cost function (14) with respect to *S* is governed by

18 $$ \begin{array}{l} \frac{\partial\tilde{J}(S)}{\partial S}=P_{s}T-\int_{0}^{T} \lambda_{1}M(t-\tau_{1})r\theta^{n}\frac{nS^{n-1}}{(\theta^{n}+S^{n})^{2}} \,dt. \end{array}  $$

According to [14], we take *k*_*m*_=10, *d*_*m*_=0.05, *k*_*p*_=1, *d*_*p*_=0.01, and set other parameter values *r*=0.02, *θ*=10, *n*=4, *P*_*s*_=1, *T*=60, *M*(0)=160, *P*(0)=10000. Then, we solve two optimal problems with these parameter values by using Matlab programs.

#### Simulation 1. Comparison of the optimal value and not optimal value about model without delay

**Fig. 4:**
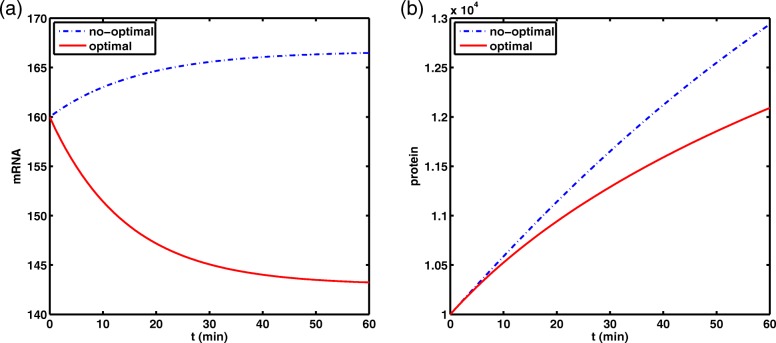
Comparison chart of the time evolution of mRNA and protein dosages of model without delay under different siRNA dosages controls. The blue dotted line corresponds to the parameter *S*=10, the red solid line corresponds to the parameter *S*=43.01

The solution obtained by the optimizer is *S*=43.01. Substituting *S* into *β*, one gets *β*=0.0199≈*r*=0.02. This shows that the degradation rate of mRNA due to RNAi has reached the maximum. When *S*>43.01, correspondingly, *β* is almost unanimously close to *r*. It implies that the amounts of protein accumulated are identical and the degradation of mRNA is subject to saturation effects when siRNA dosage is larger. Meanwhile, by employing the optimal result *S*=43.01 together with a no-optimal value *S*=10 of siRNA dosage and remaining parameters are as above, we make a comparison about the time evolution of mRNA and protein dosages of model without delay under different siRNA dosages controls (see Fig. 4).

#### Simulation 2. Comparison of the optimal value and not optimal value about model with delay

**Fig. 5:**
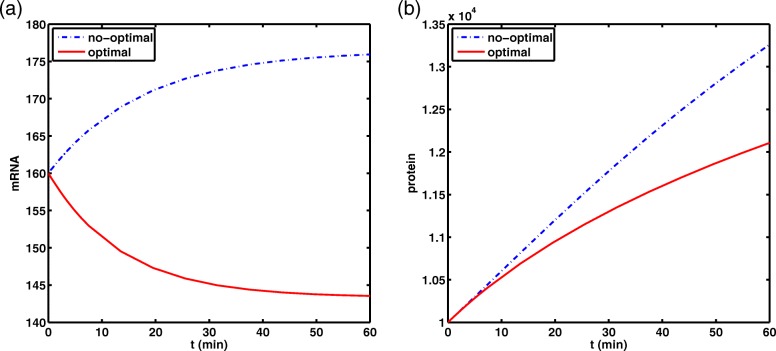
Comparison chart of the time evolution of mRNA and protein dosages of model with delay under different siRNA dosages controls. The blue dotted line corresponds to the parameter *S*=10, the red solid line corresponds to the parameter *S*=30.37

For this case, take *τ*_1_=2.5, *τ*_2_=1 and the other parameters are taken as those in simulation 1. The solution obtained by the optimizer is *S*=30.37. After substituting, one gets *β*=0.0198≈*r*=0.02. Although the two results differ by 12.64, the corresponding *β* is almost the same. This shows that the degradation rate of mRNA due to RNAi is almost the highest under the optimal conditions. Similarly, when *S*>30.37, the RNAi-mediated degradation of mRNA is subject to saturation effects, we also make a comparison like simulation 1 at *S*=30.37 and *S*=10 (see Fig. 5). In addition, Table 1 gives the value of the optimal siRNA dosage, protein accumulation (PA) and the cost function value J. Obviously, with the participation of time delays, the accumulation of protein is much lower than when there is no time delays, although both of *S* are taken at the best value.

**Table 1:** Comparison of effects with and without delays

values	with delays	without delays
S	30.37	43.01
PA	2.6199∗10^05^	1.5886∗10^07^
J	2.6381∗10^05^	1.5889∗10^07^

*#* PA: the accumulation of protein

## Discussion

What we interest in is a mathematical model that reflects the relationship between the RNAi effect and the siRNA dose, which is called the dose-effect model. The study had three primary goals. The first was to depict and forecast the evolution rules of mRNA and protein by the dynamic analysis. The second was to study the effect of parameters on periodic oscillation. The third was to explore the optimal dosage for the significant silencing efficiency. Our work provides a theoretical basis for more precise and economical RNAi experiments and applications. Even so, there are some questions worth exploring further. One is that the degradation and amplification process of siRNA should be considered in RNAi model. The second is that the stochastic effects and variable siRNA dosage should be involved in our model. These factors will result in more complicated dynamic behaviors and reveal more mechanisms of RNAi.

## Conclusions

In this paper, we reference a simple Hill kinetic model proposed by [13] and consider the potential effect of two time delays. One is degradation of mRNA due to RNAi, other one is carriage of mRNA from nucleus to cytoplasm. For the improved time-delay system, the role of time delays and the dynamic behavior of system are discussed. Qualitative analyses indicate that the introduction of time delays changes the dynamic behaviors of the system. In detail, as delays increase, the unique positive equilibrium firstly is oscillatory stable and then loses its stability via a Hopf Bifurcation. Furthermore, we give the corresponding parameter scales for these results. Meanwhile, the period of the oscillation solution shows that when the dosage of siRNA is large, oscillating periods are identical for disparate number of siRNA target sites in spite of it greatly impacts the critical siRNA dosage which is the switch of oscillating behavior. And then, parametric sensitivities of the limit cycle is determined. The results indicate that both of degradation lag and maximum degradation rate of mRNA due to RNAi are principal elements on determining periodic oscillation. After that, we propose and solve a simple optimization problem for ODEs model (19) and DDEs model (20) based on the optimization theory. The rational dosage of siRNA is given for both enhancing RNAi efficiency and reducing cost by a Matlab program. The results imply that the optimal dosage of siRNA with delay effects is less than one without time delay.

## Methods

In this section, we apply and expand the model recommended in [13]. This model well describes the mRNA and protein level in RNA interference process for different dosages of siRNA in mammalian cells in vitro, and great predicts the saturation effect observed experimentally of the RNAi process [13]. The RNAi process caused by siRNA (*S*) is encapsulated into a whole, and the degradation of the target mRNA (*M*) due to RNAi is expressed in the form of a functional reaction. In addition, the protein corresponding to the target mRNA is denoted as *P*. The time evolution of the dosages of mRNA and protein can be described by the ordinary differential equations (ODEs) as follows:

19 $$ \left\{ \begin{array}{ll} \frac{dM(t)}{dt}=k_{m}-d_{m}M(t)-\frac{rS^{n}}{\theta^{n}+S^{n}}M(t),\\ \frac{dP(t)}{dt}=k_{p}M(t)-d_{p}P(t), \end{array}\right.  $$

where *M* is transcribed at a rate *k*_*m*_ from the promoter; *d*_*m*_ and *d*_*p*_ are the degradation rates of *M* and *P*, respectively. *P* is translated at a rate *k*_*p*_ form *M*. The extra degradation rate of *M* as a result of RNAi is the third segment of the first equation of (19), which is a Hill-kinetic model. Positive integer *n* is a Hill coefficient, representing the number of siRNA bounded on the target mRNA (or the number of siRNA target sites). *r* and *θ* tie to the potency of RNAi induced by siRNA [16]: *r* denotes the maximal regression rate of *M* because of RNAi, *θ* is the dosage of *S* required to reach half of the maximal degeneration rate *r*.

Time delay plays an important role in many biological dynamical systems. There are two important biological delays that must be considered when modeling RNAi. One is the RNAi process caused by siRNA, using *τ*_1_ to describe it. The other one is the transportation process of mRNA from nucleus to cytoplasm, introducing *τ*_2_ to represent it. Then, the time evolution of the dosages of mRNA and protein can be described by the following delay differential equations (DDEs):

20 $$ \left\{ \begin{array}{ll} \frac{dM(t)}{dt}=k_{m}-d_{m}M(t)-\frac{rS^{n}}{\theta^{n}+S^{n}}M(t-\tau_{1}),\vspace{0.5em} \\ \frac{dP(t)}{dt}=k_{p}M(t-\tau_{2})-d_{p}P(t),\vspace{0.5em}\\ \end{array} \right.  $$

with the initial condition: *M*(*t*)=*M*(0) and *P*(*t*)=*P*(0) for −*max*{*τ*_1_,*τ*_2_}≤*t*≤0. It is assumed that all the parameters of model (20) are positive.

In real RNAi experiments and applications, the biological time delays are ubiquitous, such as inhibiting the expression of chitinase of migratory locust, gene knockout in animal and inhibiting cancer proliferation. Therefore, our improved time delay model is more convincing in describing the relationship between siRNA measurement and RNAi efficiency in eukaryotic cells.

## Data Availability

Data sharing is not applicable to this article as no datasets were generated or analysed during the current study.

## Abbreviations

DDEs: Delay differential equations
dsRNA: Double-stranded RNA
mRNA: Messenger RNA
ODEs: Ordinary differential equations
RISC: RNA induced silencing complex
RNAi: RNA interference
siRNA: Small interfering RNA
